# Analytical Framework for Online Calibration of Sensor Systematic Errors Under the Generic Multisensor Integration Strategy

**DOI:** 10.3390/s25103239

**Published:** 2025-05-21

**Authors:** Benjamin Brunson, Jianguo Wang

**Affiliations:** Department of Earth and Space Science and Engineering, Lassonde School of Engineering, York University, Toronto, ON M3J 1P3, Canada; jgwang@yorku.ca

**Keywords:** pre-analysis, generic multisensor integration strategy, error analysis in discrete kalman filtering, integrated kinematic positioning and navigation, online sensor calibration

## Abstract

This paper proposes an analytical framework for pre-analyzing the potential performance of online sensor calibration in Kalman filtering. Taking a multi-sensor integrated kinematic positioning and navigation system as an example, a pre-analysis of the system performance can be conducted: the observability of individual sensor systematic error states; minimum estimable values of sensor systematic error states; and minimum detectable systematic errors in sensor observations. These measures together allow for a rigorous characterization of the potential performance of a system as part of mission planning. The proposed framework enables a thorough evaluation of the relative value of different calibration maneuvers and sensor configurations before data collection by simulating the anticipated trajectory, without even requiring the construction of a physical system. When used with the Generic Multisensor Integration Strategy (GMIS), the proposed framework provides unique insight into the potential performance of IMU sensors. To illustrate the utility of the proposed framework, two situations were analyzed: one where no specific calibration maneuvers were undertaken and one where a circular motion maneuver was undertaken. The results show the potential and practicality of the proposed framework in firmly establishing best practices for field procedures and learning about the system’s capability when using online sensor calibration.

## 1. Introduction

Appropriately accounting for systematic errors in sensor measurements is paramount to any multisensor integrated positioning and navigation system, especially those used for Direct Geo-Referencing (DG) applications where biases can have a significant impact on the quality of its trajectory solution. The systematic errors of a sensor fall into two categories: intrinsic errors, which are internal to the sensor, and extrinsic errors, which have to do with the geometric relationship between the sensor and other system components. For example, the intrinsic errors in an IMU sensor include its accelerometer and gyroscope biases, scale factor errors, and axial misalignments [1,2,3,4], while the IMU extrinsic errors include the lever arm vector and/or boresight angles relating to other positioning/orientation sensors such as GNSS antennae, cameras, or LiDAR sensors, etc. [2,5,6,7,8] Ensuring the proper calibration of these errors is of the utmost importance to perform data fusion. Despite this, rigorous calibration procedures can require specialized equipment and calibration regimens and are mostly conducted offline [4]. Practical considerations can make accounting for sensor systematic errors more complicated as well. For example, IMU sensor systematic errors generally vary from turn-on to turn-on or even over the course of a single operating period [9]. Performing online sensor calibration while minimizing calibration costs is particularly desirable when using IMU sensors.

Multisensor integrated kinematic positioning and navigation systems deployed in DG technology are mainly centered around using GNSS/IMU sensors alongside visual and/or LiDAR odometry. There is a significant body of work exploring online, or in-run, calibration of an IMU’s systematic errors. IMU sensors also generally pair very well with GNSS receivers, due to the non-wandering nature of GNSS positioning solutions, which reins in IMU random walk errors. The procedures for performing online calibration of IMU sensors are well-established in the literature, but there has previously been no standardized framework for evaluating the relative value of different calibration maneuvers.

There are two primary classes of techniques that are generally applied to the online IMU calibration problem:

- Optimization-based techniques: these typically apply optimization techniques such as the Least Squares Method (LSM), Maximum Likelihood Estimation (MLE), or Kalman Filtering (KF) to estimate IMU sensor errors [4,10,11,12], which have the benefit of encoding explicit sensor error models as part of the optimization problem and allowing for a more rigorous reliability analysis as part of the online calibration process.
- Machine learning-based techniques: these typically apply Machine Learning (ML) algorithms to improve the solution quality that an IMU can provide. Several prevalent approaches have been proposed: parametric estimation of IMU systematic errors using ML, which allows for them to be modeled and accounted for via conventional means [13,14]; or denoising the IMU data to reduce standard deviations [15,16].

One of the biggest consistent challenges in online IMU calibration is in the observability of the IMU intrinsic and extrinsic errors. Many applications are ill-suited to the estimation of systematic errors related to specific sensing axes [3,11,17]. There has been an encouraging shift in the literature towards “observability-aware” systems that commonly use measures such as the observability Gramian [18] to adapt the online calibration process to what states are currently observable [7,10]. While the observability Gramian provides a clear sense of whether or not the system is observable, this measure is all-or-nothing, as it cannot distinguish between an observable system and an observable but poorly-posed system [18]. Krener and Ide [18] proposed several measures for characterizing systems as being “more” or “less” observable. Notably, a truncated Singular Value Decomposition of a Least-Squares system was used to provide reliable calibration of observable states, even in degenerate cases [7].

Current approaches to online calibration generally ignore pre-analysis environments, which can be essential in identifying potential issues and establishing best practices in the field. Directly comparing the anticipated quality of online sensor calibration over multiple candidate trajectories is particularly desirable in these environments. In particular, some machine-learning approaches to online sensor calibration are ill-suited to these environments due to their reliance on having actual sensor data to denoise [15,16].

This paper proposes a holistic, rigorous analytical framework for pre-analyzing the potential performance of online sensor calibration. The proposed framework is an optimization-based approach to online calibration that allows for a comprehensive assessment of different candidate trajectories/sensor configurations, as well as intuitive comparisons between them. The framework relies solely on the geometry of a proposed trajectory and does not require either data collection or simulation. The proposed analytical framework is an optimization-based approach using Kalman Filtering and is based upon the combined strengths of evaluating:

- The degree of observability of individual states or a group of states, after [19]. The pre-analysis of state observability allows for the determination of which sensor systematic errors are essential to model and the impact of specific calibration maneuvers on their estimation.
- The reliability in Kalman Filters after [20,21], which can deliver the minimal detectable values of the targeted constant systematic errors as outliers.
- The estimatability via Hypothesis testing of augmented sensor systematic error states in Kalman Filters after [20,21,22], which predicts how well such states can be estimated.

These three core pillars may be organically combined to intensively assess the potential performance of the augmented systematic error states being studied.

To illustrate the practicality of the proposed framework, it has been realized under the Generic Multisensor Integration Strategy (GMIS), which is documented in [23]. Under the traditional multisensor integration strategy, an IMU sensor first functions as the core sensor (mostly at the highest data rate) deployed to conduct free inertial dead reckoning successively in order to deliver a nominal navigation solution between two observation epochs from any aiding sensor, which is further used to generate error measurements against the measurements from the aiding sensor. Then, these error measurements are used to estimate the system states, including the augmented IMU’s systematic error states. The GMIS significantly deviates from the traditional integration strategy by directly employing all of the individual measurements from all constituent sensors, inclusive of the IMU measurements. The direct measurement update of individual measurements in Kalman filtering allows for the direct estimation of each constituent sensor’s systematic errors and also their measurement residuals [24].

In a similar manner to geodetic control network adjustment using Least-Squares methods, a performance pre-analysis of the positioning Kalman Filter can be conducted for a given trajectory without having any real measurements available. This paper additionally explores the application of the framework to two sets of simulated trajectories to illustrate the detailed pre-analysis that is possible under the proposed framework.

This introduction is followed by Section 2, which briefly describes the system and measurement models with the emphasis on augmenting IMU systematic errors in the state vector after the GMIS. Section 3 proposes the analytic pre-analysis framework and how it can specifically be applied to the online IMU calibration upon the models described in Section 2. Then, Section 4 goes through several testing scenarios to illustrate the proposed pre-analysis framework. These scenarios include the commonly-used circular motion calibration maneuver, as well as a test trajectory that does not include any special maneuvers for calibration at the start of the observation period. Section 5 concludes the manuscript and provides an overview of the research contributions and specific conclusions that may be drawn from this work.

## 2. Model Building After the GMIS

At its core, the GMIS’ system model is built upon 3D rigid body kinematics alongside shaping filters for augmenting the sensor systematic errors in the state vector. Additionally, all positioning and orientation sensor observations are modeled directly in the Kalman filter’s measurement models without requiring any core sensor, allowing for a generic data fusion structure of any integrated sensors. Specifically, the GMIS allows directly modeling the specific force vector and angular rate vector of any constituent IMU sensor, the GNSS observations, and observations from any other additional sensors. Refer to [23,24,25] for more details about the GMIS.

Since this research is focused on a pre-analysis of proposed trajectories, the GNSS data are loosely coupled while the IMU-specific force and angular rate observations are still integrated in a tightly-coupled manner in Kalman Filtering. In other words, the GNSS-derived positions/velocities are used instead of directly applying the GNSS observations.

### 2.1. System States

The core system states in the GMIS are:

- The 3D position, velocity, and acceleration states (9 states).
- The 3 attitude states and their rate of change (6 states).

There are different ways to model the angular motion of a system after the GMIS [25]. Here, the Roll-Pitch-Heading attitude model is selected for its ease of use and interpretation.

In addition, the system state vector is augmented with 15 additional states to model the systematic errors of an IMU through shaping filters. Specifically, the 6 accelerometer- and gyroscope-specific biases and 6 scale factor errors, as well as the 3 IMU lever arm components with respect to the GNSS antenna centre in the IMU body frame. Altogether, the system state vector is defined as

(1) $$x={\left[\begin{array}{ccc} x_{p}^{T} & x_{a}^{T} & x_{IMU}^{T} \end{array}\right]}^{T}$$

where $x_{p}={\left[\begin{array}{ccc} p^{T} & v^{T} & a^{T} \end{array}\right]}^{T}$ (position, velocity, and acceleration vectors), $x_{a}={\left[\begin{array}{cccccc} \alpha & \beta & \gamma & \dot{\alpha } & \dot{\beta } & \dot{\gamma } \end{array}\right]}^{T}$ (the system roll, pitch, and heading, along with their time derivatives), and $x_{IMU}={\left[\begin{array}{ccccc} b_{g}^{T} & s_{g}^{T} & b_{s}^{T} & s_{s}^{T} & r^{T} \end{array}\right]}^{T}$ (3 × 1 subvectors in the order of the bias and scale factor error vectors of three gyros, the bias and scale factor error vectors of three accelerometers, and the IMU 3 × 1 lever arm vector, respectively), which will be calibrated online recursively via Kalman Filtering.

### 2.2. System Model

The core of the system model is given as follows

(2) $$x_{p}\left(k+1\right)=\left[\begin{array}{ccc} I_{3} & \Delta tI_{3} & \frac{1}{2}\Delta t^{2}I_{3}\\ 0_{3} & I_{3} & \Delta tI_{3}\\ 0_{3} & 0_{3} & I_{3} \end{array}\right]x_{p}\left(k\right)+\left[\begin{array}{c} \frac{1}{6}\Delta t^{3}I_{3}\\ \frac{1}{2}\Delta t^{2}I_{3}\\ \Delta tI_{3} \end{array}\right]\dot{a}\left(k\right)=A_{p}x_{p}\left(k\right)+B_{p}w_{p}\left(k\right)$$

(3) $$x_{a}\left(k+1\right)=\left[\begin{array}{cc} I_{3} & \Delta tI_{3}\\ 0_{3} & I_{3} \end{array}\right]x_{a}\left(k\right)+\left[\begin{array}{c} \frac{1}{2}\Delta t^{2}I_{3}\\ \Delta tI_{3} \end{array}\right]w_{a}\left(k\right)=A_{a}x_{a}\left(k\right)+B_{a}w_{a}\left(k\right)$$

wherein $\left(k\right)$ denotes a quantity observed/estimated at epoch k, Δt denotes the time difference between epoch k+1 and epoch k, $I_{3}$ is a 3 × 3 identity matrix $w_{p}=\dot{a}$ and $w_{a}$ are the process noise vectors with assumed mean values of zero and associated covariance matrices of ***Q****_p_* and ***Q***_α_, respectively, under the assumption that the linear acceleration vector and the change of the attitude angles are constant over the limited small time interval between two successive time instants.

For the augmented state elements corresponding to the IMU’s bias and scale factor errors and lever arm vector, the dynamics are modeled via corresponding shaping filters. The changes in the IMU systematic error states are modeled as white process noise in the following system model:

(4) $$x_{IMU}\left(k+1\right)=x_{IMU}\left(k\right)+\Delta tw_{IMU}\left(k\right)$$

where $w_{IMU}$ is the white process noise vector and $x_{IMU}$ is the augmented state vector.

### 2.3. Observation Model

This research uses a loosely-coupled architecture to integrate GNSS observations. Consequently, the GNSS-derived position/velocity vector is given as

(5) $$z_{G}+v_{G}=\left[\begin{array}{cc} I_{6} & 0_{6,3} \end{array}\right]x_{p}\left(k+1\right)=C_{G,p}x_{p}\left(k+1\right)$$

where $z_{G}$ is the GNSS-derived position and velocity vector, $v_{G}$ is the residual vector of $z_{G}$, $C_{G,p}$ is the corresponding design matrix, and $x_{p}\left(k+1\right)$ is the Position, Velocity, and Acceleration state vector at epoch k+1 as in (2).

The IMU accelerometer and gyroscope observations were tightly-coupled in the data fusion. For the specific force vector, one has

(6) $$f_{ib}^{b}+v_{f}=C_{n}^{b}a+C_{n}^{b}g+C_{n}^{b}\left\{2\omega_{ie}^{n}+\omega_{en}^{n}\right\}\times v+C_{n}^{b}\omega_{ib}^{b}\times \omega_{ib}^{b}\times r_{i}-b_{a}-S_{a}f_{ib}^{b}$$

where $f_{ib}^{b}$ denotes the specific force vector acquired by the three accelerometers with respect to the inertial frame in the body frame, $v_{f}$ is the residual vector of $f_{ib}^{b}$, $C_{n}^{b}$ denotes the rotation matrix from the navigation frame to the body frame, a denotes the acceleration vector of the body frame in the navigation frame at epoch k+1, g denotes the local gravity vector, expressed in the navigation frame, v denotes the velocity vector of the body frame in the navigation frame, r denotes the lever arm vector for the IMU in the body frame, $b_{a}$ denotes the accelerometer bias vector, and $S_{a}$ denotes the accelerometer scale factor error matrix. As a general rule, $S_{a}$ is a fully populated 3 × 3 matrix, but the off-diagonal cross-coupling errors are often neglected in practice, as they are in this research.

The gyroscope (angular rate) measurement model is given as follows

(7) $$\omega_{ib}^{b}+v_{\omega }=\omega_{nb}^{b}-b_{g}-S_{g}\omega_{ib}^{b}+C_{n}^{b}\left(\omega_{ie}^{n}+\omega_{en}^{n}\right)$$

where $\omega_{ib}^{b}$ denotes the angular rate vector of the body frame with respect to the inertial frame in the body frame acquired by the three gyros, $v_{\omega }$ denotes the residual vector of $\omega_{ib}^{b}$, $\omega_{nb}^{b}$ denotes the angular rate vector of the body frame with respect to the navigation frame in the body frame, $\omega_{ie}^{n}$ denotes the angular velocity vector of Earth’s self-rotation with respect to the inertial frame in the local navigation frame, $\omega_{en}^{n}$ denotes the angular velocity vector of the navigation frame with respect to Earth in the local navigation frame, $b_{g}$ denotes the gyroscope bias vector, and $S_{g}$ denotes the gyroscope scale factor error matrix.

The partial derivatives of (6) with respect to the individual linear motion states, angular motion states, and augmented states construct the following Jacobian matrices:

(8) $$C_{s,p}=\left[\begin{array}{cc} 0_{3,6} & C_{n}^{b} \end{array}\right]$$

$$C_{s,a}=\left[\begin{array}{cc} \frac{\partial C_{n}^{b}}{\partial \alpha }\left(a+g+\left\{2\omega_{ie}^{n}+\omega_{en}^{n}\right\}\times v+\omega_{ib}^{b}\times \omega_{ib}^{b}\times r\right) & \frac{\partial C_{n}^{b}}{\partial \beta }\left(a+g+\left\{2\omega_{ie}^{n}+\omega_{en}^{n}\right\}\times v+\omega_{ib}^{b}\times \omega_{ib}^{b}\times r\right) \end{array}\right.$$

(9) $$\left.\begin{array}{cc} \frac{\partial C_{n}^{b}}{\partial \gamma }\left(a+g+\left\{2\omega_{ie}^{n}+\omega_{en}^{n}\right\}\times v+\omega_{ib}^{b}\times \omega_{ib}^{b}\times r\right) & 0_{3} \end{array}\right]$$

(10) $$C_{s,IMU}=\left[\begin{array}{ccc} 0_{3,6} & I_{3} & \begin{array}{cc} \begin{array}{ccc} f_{ib,\;measx}^{b} & 0 & 0\\ 0 & f_{ib,\;measy}^{b} & 0\\ 0 & 0 & f_{ib,\;measz}^{b} \end{array} & \left[C_{n}^{b}\omega_{ib}^{b}\times \omega_{ib}^{b}\right] \end{array} \end{array}\right]$$

After [26], the Jacobian matrices of the angular rate observations in (7) are given as

(11) $$C_{g,a}=\left[\begin{array}{cc} 0_{3} & \begin{array}{ccc} 0 & \cos\alpha & \sin\alpha \cos\beta \\ 1 & -\sin\beta & 0\\ 0 & \sin\alpha & -\cos\alpha \cos\beta \end{array} \end{array}\right]$$

(12) $$C_{g,IMU}=\left[\begin{array}{ccccc} I_{3} & \begin{array}{ccc} \omega_{ib,measx}^{b} & 0 & 0\\ 0 & \omega_{ib,measy}^{b} & 0\\ 0 & 0 & \omega_{ib,measz}^{b} \end{array} & 0_{3} & 0_{3} & 0_{3} \end{array}\right]$$

### 2.4. Using the Discrete Kalman Filter for Pre-Analysis

Using the system model in Section 2.2 and observation model in Section 2.3, the a priori covariance matrices of the observation and process noise vectors and the initial state vector along with its a priori covariance matrix may be propagated through a predefined trajectory without using any actual observations. The following information is required for this error propagation:

- The three-dimensional positions, velocities, and accelerations.
- The attitude angles and their time derivatives.
- The anticipated covariance matrices of the observation and process noise vectors.
- The initial state vector with its a priori covariance matrix

Once the mentioned information is made available from a simulated trajectory, one can generate the system state transition matrix A, process noise transition matrix B, and design matrix C, enabling an epoch-wise pre-analysis via Kalman filtering. Conceptually, this pre-analysis is the same as how a Least-Squares problem is pre-analyzed before the acquisition of measurements takes place, and this pre-analysis may be conducted using the standard Kalman Filter error propagation, as detailed in [27].

## 3. Analytical Framework for Online Sensor Calibration

From a design or pre-analysis perspective, it is valuable to evaluate the anticipated performance of an online calibration procedure in Kalman filtering. Although the existing research lacks an exploration of this essential design stage, there is an increasing number of related studies being conducted, as in [7,28].

This section is focused on presenting an analytical framework for online sensor calibration in a multisensor integrated kinematic positioning and navigation system from the following aspects:

1. Characterizing the observability of the augmented sensor systematic error states to further evaluate the feasibility of specific maneuvers and the relative merits of including specific systematic error estimates in the state vector (Section 3.1).
2. Determining the minimum estimable values of the sensor systematic error states to confirm how well they can be significantly identified in comparison with their anticipated magnitudes after the principles of internal reliability in Kalman Filtering [21] (Section 3.2).
3. Optimizing the anticipated trajectory designs to produce the best-performing candidates. (Section 3.3).
4. Performing analysis of the redundancy contribution of the observations in a designed trajectory to identify potential issues (Section 3.4).

Following the proposed framework in Section 3.1, Section 3.2, Section 3.3 and Section 3.4, a workflow is presented for the overall framework in Section 3.5.

### 3.1. Observability and Degree of Observability of States

The observability of system states is an important quality measure in a linear system. Generally speaking, a system is completely observable over a specific time span $\left(t_{0},\;t_{k}\right)$ if and only if the initial system state vector at $t_{0}$ can be determined from the observations collected over the given interval. A system is also differentially observable if it is observable over every sub-interval over $\left(t_{0},\;t_{k}\right)$. A system is instantaneously observable if it is completely observable over the interval $\left(t_{k},t_{k+1}\right)$ [29].

It is simpler to identify systems that are instantaneously observable than systems that are differentially or completely observable. A system is differentially observable if and only if it is instantaneously observable for every observation epoch in the interval $\left(t_{0},\;t_{k}\right)$ [29]. A system is completely observable over an interval if it is also differentially observable over that interval, although this is not a necessary condition [29].

Although complete, differential, and instantaneous observabilities are essential properties in characterizing a linear system, they also have inherent limitations in that they fall into all-or-nothing criteria and cannot quantitatively express which states are more observable than the others among the observable states. For example, the observation quality does not factor into system observability, so it is possible to have a system that is completely observable due to the observation geometry but is practically unobservable because the available observations are not accurate enough to reliably determine the system states. Given these limitations, it is valuable to establish the “degree of observability” of individual states in a Kalman Filter. There are many ways to characterize the degree of observability, some of which even include observation accuracy as part of their criteria. This subsection specifically describes three measures of observability: the observability index, which does not identify degree of observability, and the observability Gramian and eigenvalues of the state covariance matrix, which do.

#### 3.1.1. The Observability Matrix

The local unobservability index is found via the observability matrix [29]

(13) $$O\left(t\right)=\left[\begin{array}{c} N_{0}\\ N_{1}\\ \vdots \\ N_{n-1} \end{array}\right]$$

wherein $N_{0}=C$ where C represents the design matrix, $N_{i}=N_{i-1}A+\frac{d}{dt}N_{i-1}$ where A represents the state transition matrix, and n is the dimension of the state vector. This matrix has $\left(n-1\right)m$ rows, where m is the number of observations at the current observation epoch, and n columns.

If the rank of (13) is equal to n, the system is instantaneously observable. If its rank is always equal to n over a specific time interval, then the system is said to be differentially observable over that interval. Furthermore, if its rank is equal to n for any epoch on an interval, the system is said to be completely observable over that interval, although a system can be completely observable without this being the case.

#### 3.1.2. The Observability Gramian

The observability of a system can be characterized by estimating the impact a true error in an element in the state vector has on the overall observation vector [18]. If a single element of the state vector $x_{i}$ has an associated disturbance of ϵ, the change in the overall vector x is given by $dx^{+i}=\epsilon e_{i}$ or $dx^{-i}=-\epsilon e_{i}$, where $e_{i}$ is the ith basis vector. The resultant impact on the observation vector is determined as follows

(14) $$z^{\pm i}=Cdx^{\pm i}$$

where the superscript “±” indicates two potential values: one corresponding with $dx^{+i}$, and one corresponding with $dx^{-i}$.

(14) can be used to populate the empirical local observability Gramian, which is defined to be a matrix P such that

(15) $$P_{ij}=\frac{1}{4\epsilon^{2}}\int_{t_{0}}^{t_{k}}{\left(z^{+i}-z^{-i}\right)}^{T}\left(z^{+j}-z^{-j}\right)dt$$

for each element $\left(i,j\right)$ of the observability Gramian.

This observability Gramian is generally evaluated over an interval but may technically be estimated at an individual observation epoch. The longer the interval that is used to estimate the observability Gramian, the better information it provides regarding the overall observability of the system. In this paper, the observability Gramian is always evaluated over the entire candidate trajectory.

The observability Gramian may be used to determine several measures of observability: the reciprocal of the smallest eigenvalue of P is the local unobservability index, with larger values of this quantity indicating a less observable system; and the ratio of the square root of the largest to the square root of the smallest eigenvalue of P is the local estimation condition number, with larger values indicating poorly-posed systems.

#### 3.1.3. Observability After Eigenvalues of the State Covariance Matrix

The eigenvalues and eigenvectors of the state covariance matrix may be used to characterize the degree of observability of linear combinations of system states [19]. Generally, the largest eigenvalue corresponds to the linear combination of states with the lowest observability, and the smallest eigenvalue corresponds to the linear combination of states with the highest observability. Analyzing the eigenvalues and eigenvectors of the state covariance matrix accounts for the impact of not only the observation geometry but also the variances of the observations on the observability of the system.

The significance of the eigenvalues/eigenvectors of a covariance matrix is not easily interpreted on its own. Consequently, the covariance matrix will be normalized prior to its eigen analysis. The normalization adopted here makes use of the diagonal matrix $D_{x}\left(0\right)$ constructed with the initial state vector variances and is calculated as follows [19]

(16) $$D_{x}^{\prime }\left(k\right)={\left(\sqrt{D_{x}\left(0\right)}\right)}^{-1}D_{x}\left(k\right){\left(\sqrt{D_{x}\left(0\right)}\right)}^{-1}$$

where $\sqrt{M}$ means the square root of the diagonal matrix, $D_{x}\left(k\right)$ is the state covariance matrix at epoch k, and $D_{x}^{\prime }(k)$ is the normalized state covariance matrix at epoch k. Further, the normalized covariance matrix in (16) is scaled using its own trace as follows

(17) $$D_{x}^{N}\left(k\right)=\frac{n}{tr\left(D_{x}^{\prime }\left(k\right)\right)}D_{x}^{\prime }\left(k\right)$$

which ensures that the eigenvalues lie between 0 and n, to simplify their interpretation.

The eigenvalue-based observability analysis from the observability Gramian and the state covariance matrix is difficult to plot or interpret, since both eigenvalues and eigenvectors are liable to change through time. In practice, one may make some concessions on such observability measures to more clearly express the evolution of the observability of specific states through time.

Specifically, neglecting the correlation between states (i.e., ignoring the off-diagonal elements of the observability Gramian or state covariance matrix) results in the corresponding eigenvectors simply being a set of n-dimensional basis vectors, with the corresponding eigenvectors being the diagonal elements of the involved matrix.

For the local observability Gramian, neglecting the correlation between states in (15) results in the following integral

(18) $$P_{ii}=\frac{1}{4\epsilon^{2}}\int_{t_{0}}^{t_{k}}{\left(z^{+i}-z^{-i}\right)}^{T}\left(z^{+i}-z^{-i}\right)dt$$

to approximate the observability of the *ith* state $\forall i\in \left(1,\;2,\;\ldots ,\;n\right)$. They can also potentially be used to approximate the local estimation condition number and local unobservability index, with an associated loss of accuracy.

For the state covariance matrix, neglecting the correlation between states in (17) results in the normalized element

(19) $${\left(D_{x}^{N}\right)}_{ii}=\frac{n}{tr\left(D_{x}^{\prime }\right)}{\left(D_{x}^{\prime }\right)}_{ii}$$

to approximate the unobservability of state element i.

### 3.2. Minimum Estimable Values of Systematic Error States

Modeling systematic errors in measurements is only meaningful if the corresponding augmented states are significantly estimable in Kalman Filtering during the online calibration process. Practically, their minimum estimable values (i.e., their significance from zero) over a specific portion of or an entire kinematic trajectory depend on their standard errors.

#### 3.2.1. Hypothesis Testing of Individual Augmented Systematic Error States

The epoch-wise covariance matrix for the system states in (14) estimates the accuracy of the state vector. Specifically, the square root of each diagonal element in $P\left(k+1\right)$ is the anticipated standard deviation of its corresponding system state [20,22].

(20) $$\sigma_{x_{i}}=\sqrt{P_{ii}\left(k+1\right)},\;i\in \left(1,\;2,\;\ldots ,\;n\right)$$

When evaluating the viability of a systematic error model or of a trajectory for online sensor error calibration, the estimate ${\hat{x}}_{i}\left(k+1\right)$ of a systematic error state $x_{i}\left(k+1\right)$ needs to be statistically significantly different from zero, which is formulated via the following null hypothesis $H_{0}$ and its alternative hypothesis $H_{a}$:

(21) $$H_{0}:{\tilde{x}}_{i}=0,\;H_{a}:{\tilde{x}}_{i}\neq 0,\;i\in \left(1,\;2,\;\ldots ,\;n\right)$$

Under the assumption that the measurements and process noises conform to normal distributions, the test statistic for $x_{i}\left(k+1\right)\;$ is

(22) $$z_{i}=\frac{{\hat{x}}_{i}}{\sigma_{x_{i}}}\sim N\left(0,\;1\right)$$

When using measurements, the test statistic is compared to a critical value $z_{crit}$ after the standard normal distribution at the specified test significance. If it satisfies

(23) $$\frac{\left|{\hat{x}}_{i}\right|}{\sigma_{x_{i}}}\geq z_{crit}$$

the null hypothesis is statistically rejected, and the alternative hypothesis is accepted at the specified test significance level.

In system design without using measurements, (23) is used to determine a minimum estimable value for ${\hat{x}}_{i}$ as follows

(24) $$\left|{\hat{x}}_{i}\right|\geq \sigma_{x_{i}}z_{crit}$$

wherein $z_{crit}$ is a critical value determined by using the specified test significance level α (Type I Error) and power 1−β (Type II Error) [21]. The minimum estimable error provides insight into the potential performance of the online sensor calibration, and as long as the minimum estimable value of ${\hat{x}}_{i}$ satisfies the design specification, it should be included in the state vector.

#### 3.2.2. Hypothesis Testing of a Group of Augmented Systematic Error States

Generally, the estimate of the augmented system states for modeling the sensor systematic errors are correlated with one another in Kalman Filtering. A hypothesis test for a group of the correlated states can be introduced analogously to the deformation analysis in surveying [20]. Usually, as a part of the statistical tests, hypothesis testing of a group of the correlated states is conducted first (as global or regional tests), and then hypothesis testing of each individual state of interest follows (as local tests). Generally, complications arise when coordinating the significance levels for tests on a group of states, as opposed to individual states, to ensure that results remain consistent [20].

Straightforwardly, a multidimensional linear hypothesis testing of a group of correlated systematic error states of interest, $x_{g}\left(k+1\right)$ is expressed through a null hypothesis and its alternative as follows

(25) $$H_{0}:H\left(k+1\right){\tilde{x}}_{g}=0,H_{a}:H\left(k+1\right){\tilde{x}}_{g}\neq 0$$

Assuming the multistates that are being tested follow a multivariate normal distribution with a zero mean vector and its covariance matrix, the test statistic corresponding to (25) is given by

(26) $$\chi_{g}^{2}={\hat{x}}_{i}^{T}H^{T}\left(k+1\right){\left(H\left(k+1\right)P\left(k+1\right)H^{T}\left(k+1\right)\right)}^{-1}H\left(k+1\right){\hat{x}}_{g}\cdot h$$

which conforms to a chi-squared distribution with *h* degrees of freedom. If data were available, this test statistic could be evaluated and compared to the critical value of the chi-squared distribution for a specified test significance/power. To accept the null hypothesis, the following condition would need to be satisfied:

(27) $${\hat{x}}_{g}^{T}H^{T}{\left(k+1\right)\left(H\left(k+1\right)P\left(k+1\right)H^{T}\left(k+1\right)\right)}^{-1}H\left(k+1\right){\hat{x}}_{g}\cdot h\leq \chi_{crit}^{2}$$

Since this work is focused on pre-analysis, the special case where ${\hat{x}}_{g}$ is an eigenvector of $H^{T}\left(k+1\right){\left(H\left(k+1\right)P{\left(k+1\right)H}^{T}\left(k+1\right)\right)}^{-1}H\left(k+1\right)$ is considered:

(28) $${\hat{x}}_{g}^{T}H^{T}{\left(k+1\right)\left(H\left(k+1\right)P\left(k+1\right)H^{T}\left(k+1\right)\right)}^{-1}H\left(k+1\right){\hat{x}}_{g}={\hat{x}}_{g}^{T}{\hat{x}}_{g}\lambda =\lambda {\left|{\hat{x}}_{g}\right|}^{2}$$

Depending upon the choice of eigenvalues, the quadratic form in (28) either represents a best-case or worst-case scenario for the statistical test. Rearranging (28) to isolate $\left|{\hat{x}}_{i}\right|$ yields

(29) $$\left|{\hat{x}}_{g}\right|\leq \sqrt{\frac{\chi_{crit}^{2}}{h\lambda }}$$

Here, the largest value of λ provides the strictest statistical test.

When the null hypothesis is instead framed as

(30) $$H_{0}:H{\tilde{x}}_{i}\neq 0,H_{a}:H{\tilde{x}}_{i}=0$$

the condition in (29) instead becomes

(31) $$\left|{\hat{x}}_{g}\right|\geq \;\sqrt{\frac{\chi_{crit}^{2}}{h\lambda }}$$

and the smallest value of λ provides the strictest statistical test.

Specifically, a hypothesis test for the significance of a group of augmented system states is a special case of (25) where $H\left(k+1\right)=I$, leading to the null hypothesis and associated alternative hypothesis of

(32) $$H_{0}:{\tilde{x}}_{i}\neq 0,H_{a}:{\tilde{x}}_{i}=0$$

and the same condition as (31) to define the minimum estimable length of the system state subvector. Here, the value of λ is given by the smallest eigenvalue of $P^{-1}$.

### 3.3. Directly Comparing Covariance Matrices

To improve the performance of online sensor calibration in the design phase, it is useful to be able to directly compare covariance matrices estimated over different trajectories to determine what provides the “most accurate” results. The simplest method of accomplishing this is by directly comparing the anticipated standard deviations for each system state element between each candidate solution, but this approach neglects the correlation between system state elements.

A covariance matrix $D_{a}$ may be considered to be “better” than another covariance matrix $D_{b}$ of a random vector when their quadratic forms satisfy [20]

(33) $$f^{T}D_{a}f\leq f^{T}D_{b}f\forall f\in R^{n}$$

or alternatively,

(34) $$f^{T}\left(D_{a}-D_{b}\right)f\leq 0\;\forall f\in R^{n}$$

The condition in (34) is satisfied when $D_{a}-D_{b}$ is a negative definite or negative semi-definite matrix, and $D_{a}$ may be considered “better” than $D_{b}$.

Of course, there are many circumstances under which $D_{a}-D_{b}$ will be an indefinite matrix. This corresponds to a situation where some portions of $D_{a}$ outperform $D_{b}$ and other portions underperform compared to $D_{b}$. Under this situation, it is useful to define a measure that estimates the degree to which $D_{a}$ is better than $D_{b}$. This research proposes a measure based on the estimated eigenvalues of $D_{a}-D_{b}$:

(35) $$c_{ab}=\frac{\sum_{i=1}^{n}\lambda_{i}}{\sum_{i=1}^{n}\left|\lambda_{i}\right|}$$

The comparison measure in (35) is a real number that is bounded between −1 and 1. When all of the eigenvalues are positive, $c_{ab}=1$ and $D_{a}$ may be considered “better” than $D_{b}$ for all states. When all of the eigenvalues are negative, $c_{ab}=-1$ and $D_{b}$ may be considered to outperform $D_{a}$ for all states. When $c_{ab}$ is positive, $D_{a}$ has some elements that perform “worse” than $D_{b}$, but $D_{a}$ may be considered “better” on the whole.

### 3.4. Minimum Detectable Values of Systematic Errors as Outliers After the Internal Reliability

On the grounds of the internal reliability in pre-analysis in Kalman Filtering [22], the redundancy indices are retrieved from the following idempotent matrix

(36) $$R_{l}=D_{v_{l}}R^{-1}$$

for the observation vector and

(37) $$R_{w}=D_{v_{w}}Q^{-1}$$

for the process noise vector, wherein $D_{v_{l}}$ and $D_{v_{w}}$ are the covariance matrices for the observation and process noise residuals, respectively.

The diagonal elements of the matrices in (36) and (37) provide the redundant indices for each of the independent observations and process noise factors, respectively, such that $r_{l_{i}}={\left(R_{l}\right)}_{ii}$ and $r_{w_{i}}={\left(R_{w}\right)}_{ii}$. $r_{l_{i}}$ and $r_{w_{i}}$ describe how much redundant information is held by the individual elements in the measurement vector and in the process noise vector. A redundancy value of 0 indicates no redundant information (equivalent to high leverage or full necessity), and a value of 1 indicates completely redundant information (either its standard error is too large or the element is geometrically irrelevant). Generally, the redundant indices should have intermediate values or be evenly distributed, as their total is equal to the total number of redundant measurements.

In the case that there is a constant bias in a specific measurement series, the constant bias may be occasionally identified epoch-wise as an outlier when it is not augmented in the state vector through a shaping filter. An analysis of its internal reliability may be able epoch-wise to determine its minimum detectable value for a specific test significance/power. As long as the bias conforms to a random constant, it may later be estimated via a shaping filter.

With this pre-analysis framework, it is possible to assess the minimum detectable constant errors in a positioning sensor by evaluating the attainable precision in the associated residual vector. This is accomplished via the relationship between the true error vector ε and the associated residual vector v

(38) $$v=-\left(D_{v}D_{l}^{-1}\right)\varepsilon =-R_{l}\varepsilon$$

where $D_{l}$ is the covariance matrix describing the *a priori* stochastic properties of the associated observation vector.

Considering only the effects on each residual element from varying its associated observation error allows (38) to be given as

(39) $$v_{i}=-r_{l_{i}}\varepsilon_{i},\;i\in \left(1,\;2,\;\ldots ,\;m\right)$$

and propagating the error from $\varepsilon_{i}$ to $v_{i}$ yields

(40) $$\sigma_{v_{i}}=\sqrt{r_{l_{i}}}\sigma_{l_{i}}$$

Assuming that a specific sensor systematic error is characterized as constant throughout its entire measurement series, it is possible to detect it successively as an outlier. The significance of the mean residual value over a time period of s observation epochs is tested using the following test statistic [22]:

(41) $$\delta_{i}\left(\alpha ,\;1-\beta \right)=\frac{\sum r_{l_{j}}}{\sigma_{l_{i}}\sqrt{s}}\varepsilon_{i}$$

under the specified significance level of α (Type I Error) and power of 1−β (Type II Error), from which the minimum detectable error is determined as

(42) $$\varepsilon_{i,min}=\delta_{i,crit}\left(\alpha ,\;1-\beta \right)\frac{\sum \sqrt{r_{l_{j}}}}{\sigma_{l_{i}}\;\sqrt{s}}$$

The minimum detectable error in (42) is used to estimate the potential detectable value of the constant systematic errors in the observation vector. It is worth noting that the estimated minimum detectable error presents a measure of the system’s potential precision rather than of the system’s potential accuracy. In other words, it estimates the minimum consistent error in the observations/process noises that could produce a detectable output through the Kalman Filter.

### 3.5. Workflow of the Analytical Pre-Analysis Framework

**Figure 1:**
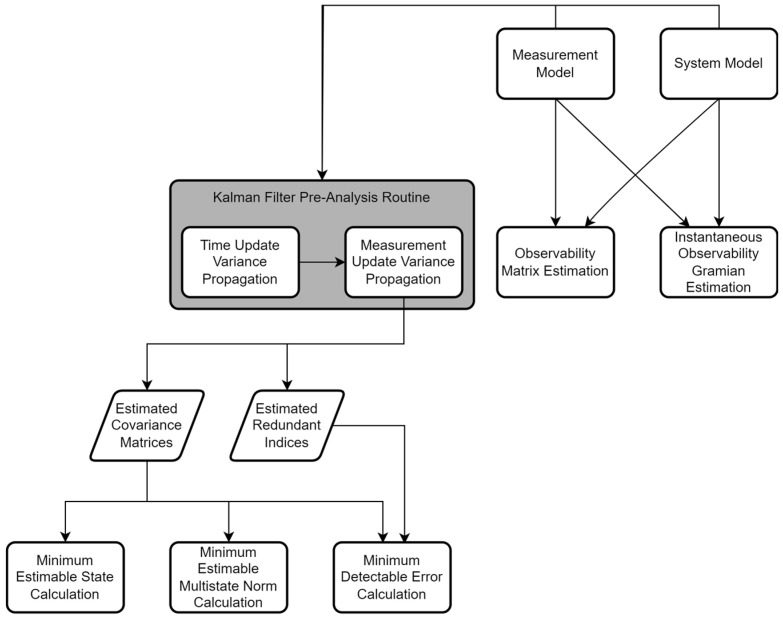
Workflow for estimating the specific quantities used in the proposed framework using the measurement models and output of the Kalman Filter pre-analysis routine.

A workflow diagram for the proposed pre-analysis framework is presented in Figure 1. The observability matrix and instantaneous observability Gramian are notably separate from the other quantities that are estimated as a part of the pre-analysis. This is because these quantities are defined purely from the observation and system models and do not rely on the Kalman Filtering error propagation.

## 4. Case Studies

The pre-analysis framework for online sensor calibration proposed in Section 3 allows for a comparative assessment of different candidate trajectories to evaluate how well they are suited for the online calibration of an IMU sensor and can quantitatively compare the calibration performance of different realizations of the same basic calibration maneuver. To practically apply this framework and illustrate its utility, two scenarios are considered in this research:

1. Case #1: No intentional calibration maneuvers. Here, we pre-analyze a trajectory derived from a real dataset without any special intention of calibration maneuvers to demonstrate how the proposed framework is practically implemented.
2. Case #2: Circular motion. A common maneuver is considered here, in which a ground vehicle travels in circles for a certain period of time. The pre-analysis is focused on how different factors, such as the average vehicle speed and the radius of circular motion, impact the online calibration.

These two case studies are explored in more detail in the subsequent sections. Moreover, both of these scenarios use the same technical specifications for all constituent positioning/orientation sensors. Table 1 summarizes the anticipated accuracies of the applied measurements. All statistical tests in this section use a significance level of α=0.01 and a power level of β=0.80 unless stated otherwise.

Additionally, both test cases assume the use of the same roof-mounted system consisting of one GNSS receiver and one MEMS IMU attached to the roof of a car. The IMU sensing axes were aligned to the vehicle frame, and the navigation frame is defined using the local East-North-Up (ENU) directions. Using a roof-mounted system mitigates the impact of vehicle vibrations, since the system is mounted above the vehicle’s suspension.

### 4.1. Case #1: No Calibration Maneuvers

**Figure 2:**
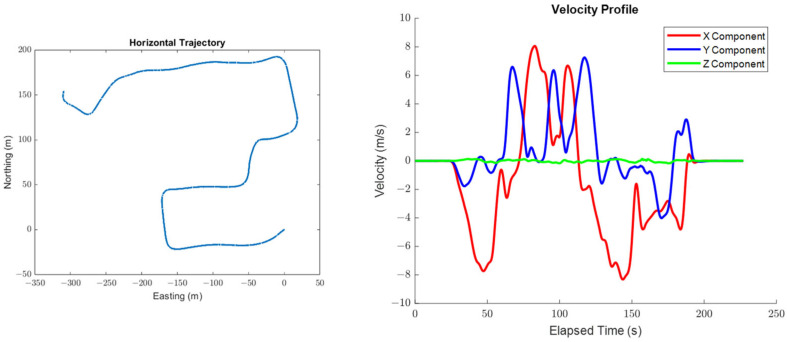
The top-down view of the trajectory used in Case #1 (**left**) and the velocity profile of the trajectory used in Case #1 (**right**).

**Figure 3:**
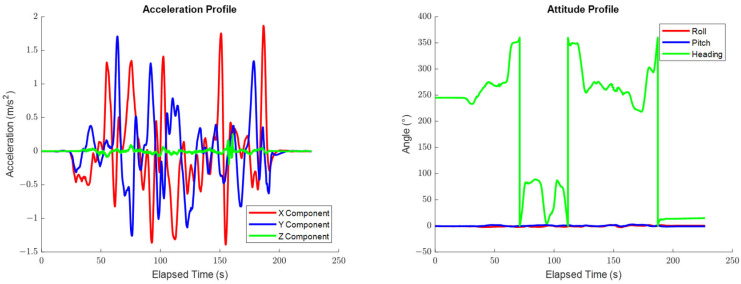
The acceleration profile of the trajectory used in Case #1 (**left**) and the attitude profile of the trajectory used in Case #1 (**right**).

Figure 2 shows the top-down view of the trajectory that was adapted from a real road dataset, as well as the trajectory’s associated velocity profile. Figure 3 shows the trajectory’s associated acceleration and attitude profiles. All calculations in this section were performed using only information derived from the positioning system’s trajectory, unless indicated otherwise.

#### 4.1.1. Observability Analysis

One major challenge in online sensor calibration, particularly for MEMS IMUs, is the observability of their associated systematic error states. Most practical applications have some implicit constraints on the vehicle’s motion; for example, ground vehicles generally do not naturally move vertically or significantly change their roll and pitch angles. As a result, most datasets have some sensor axes associated with practically unobservable systematic errors. While unobservable systematic error states may be included in the Kalman Filter without introducing numerical instability into the state estimation process, their inclusion as estimated states is unnecessary and increases the computational load of the online calibration process.

The test trajectory used in this section is defined for a car traveling on a relatively flat road and therefore has no significant vertical motion or roll/pitch dynamics, as can be observed in Figure 2 and Figure 3. The systematic errors relating to the vertical system dynamics or the dynamics in the roll and pitch are therefore intuitively expected to be unobservable in practice.

The rank of the observability matrix calculated via (13) for each observation epoch over the entire dataset was 22 for the 30 states that are being estimated. It may therefore be inferred that there are 8 unobservable states for this dataset, although there is no indication of what states are not observable.

Estimating the observability Gramian over the entire dataset yields an unobservability index of 133,172 and an estimation condition number of 2,972,916,621. The large magnitude of these numbers suggests that some systematic error states are completely unobservable over this dataset. The unobservability index and estimation condition number are valuable measures of overall observability, but they are also difficult to interpret since they are unbounded estimates, and there is subjectivity in the definition of what constitutes a “large” issue with system observability.

To identify which states are unobservable, the eigenvalues and eigenvectors of the normalized state covariance matrix are estimated. Since eight states were determined to be unobservable from the rank of the observability matrix, the eight eigenvectors with the largest associated eigenvalues describe the systematic error states that are least observable. Table 2 presents an overview of the eight largest eigenvalues of the normalized state covariance matrix and their associated eigenvectors.

The eigenvectors with the largest associated eigenvalues indicate the quantities that are the least observable. These include the IMU lever arm X, Y, and Z components, the X and Y components of the gyroscope scale factor errors, and the X, Y, and Z components of the accelerometer scale factor error. The elements contributing the most to the lack of observability of the system are the lever arm X and Y components. Note that there is often repetition in the dominant components of the eigenvectors presented in Table 2. For example, the X and Y components of the gyroscope scale factor error are dominant in two of the eigenvectors. This indicates that there is generally poor observability of horizontal gyroscope scale factor errors, as opposed to this poor observability being limited to a single direction in the horizontal plane.

The eigenvalues and eigenvectors in Table 2 were evaluated for a single state covariance matrix at the end of the trajectory. The eigenvector analysis provides a clear indication of what states are lacking observability in the system, but the evolution of the dominant components of the eigenvectors through time is challenging to visualize and interpret.

**Figure 4:**
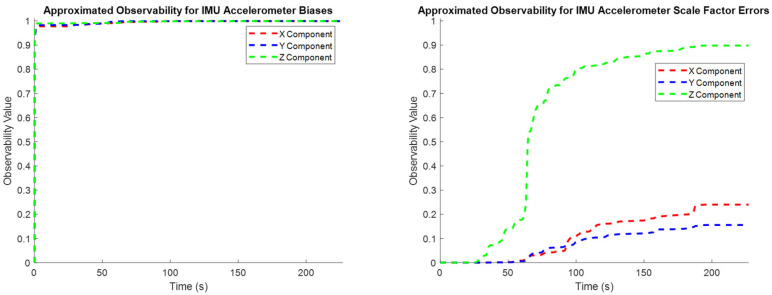
The observability of the systematic errors in the accelerometers (**left**: biases; **right**: scale factor errors).

**Figure 5:**
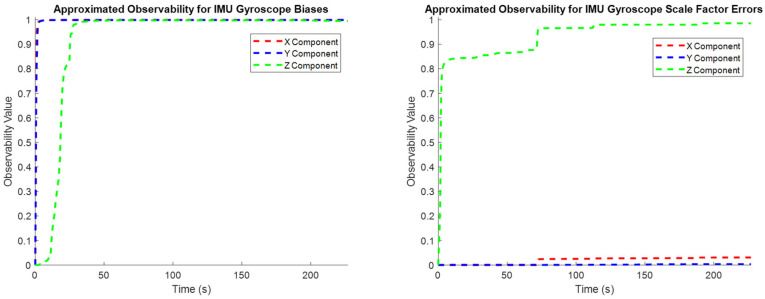
The observability of the systematic errors in the gyroscopes (**left**: biases; **right**: scale factor errors).

**Figure 6:**
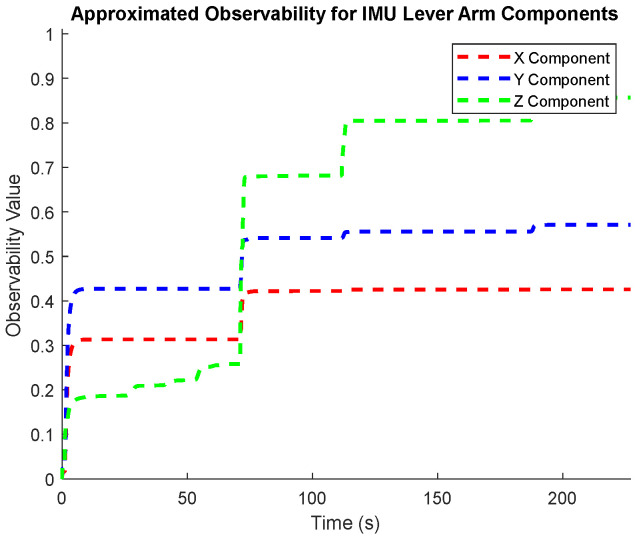
The observability of the lever arm components.

To characterize how the observability of individual states evolves through time, the normalized covariance elements in (19) are used to approximate the observability index for individual state elements throughout the entire test trajectory (Figure 4, Figure 5 and Figure 6). These plots confirm the intuitive conclusions that the lever arm X and Y components and the augmented sensor scale factor error states are generally less observable than the other systematic error states over the test trajectory. This generally agrees with the results presented in Table 2, although the more rigorous eigenvector analysis does additionally identify the Z component of the lever arm vector to be less observable.

#### 4.1.2. Minimum Estimable Systematic Error States

**Figure 7:**
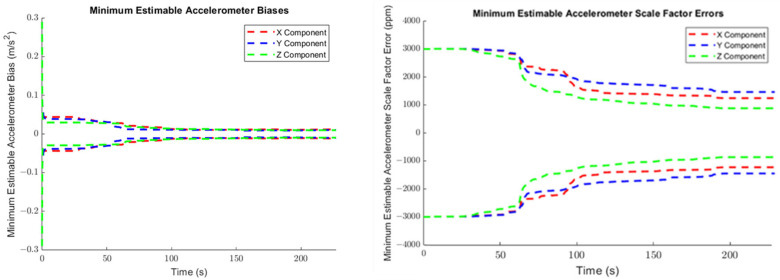
The minimum estimable systematic errors in the accelerometers (**left**: biases; **right**: scale factor errors).

**Figure 8:**
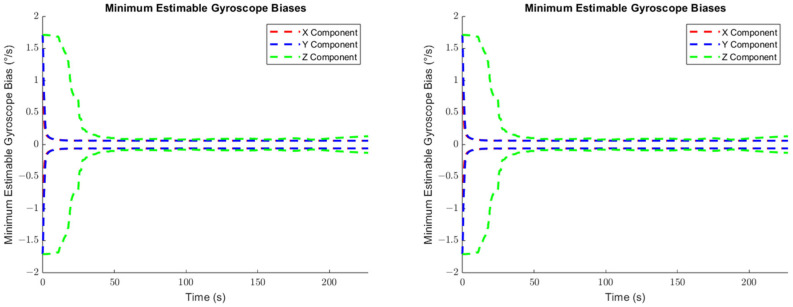
The minimum estimable systematic errors in the gyroscopes (**left**: biases; **right**: scale factor errors).

**Figure 9:**
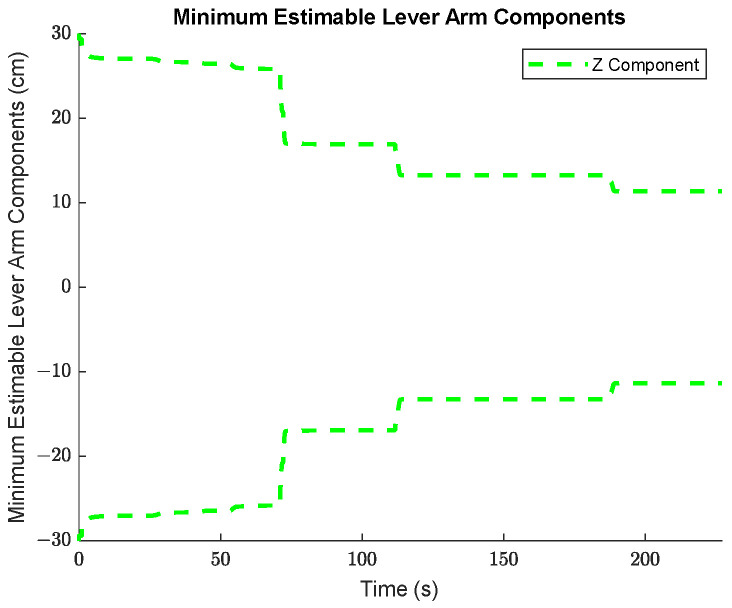
The minimum estimable lever arm components for the trajectory.

The minimum estimable values of the augmented systematic error states, based on (24) in Section 3.2.1 are plotted for the accelerometers in Figure 7, for the gyroscopes in Figure 8, and for the lever arm vector between the IMU sensor and the GNSS receiver in Figure 9.

As can be seen in Figure 7 and Figure 8, the propagated standard errors of the IMU sensor bias states clearly have converged quickly and remained stable until the end of the dataset, except for the bias associated with the Z-gyroscope, which took approximately 20 s longer to converge, as the Z-gyroscope was vertically-aligned and could not be oriented while the vehicle was stationary. However, the estimate of the Z-gyroscope bias converged quickly once the system began to move.

The trajectory in Case #1 did not experience high accelerations. The accelerometers therefore did not experience the high dynamics that would have produced larger associated scale factor errors. This means that, although Figure 7 exhibits an improvement in the estimated scale factor error states of the three accelerometers through time, this improvement is very limited over the entire trajectory.

The minimum estimable values of the X- and Y-gyroscope scale factor error states barely changed at all over the whole trajectory (refer to Figure 8) as the trajectory is from a land vehicle and therefore does not have significant dynamics in its pitch and roll, which in turn restricts how estimable the augmented scale factor error states associated with the X- and Y-gyroscopes are. Apparently, the augmented scale factor error state associated with the Z-gyroscope could be estimated to an accuracy of ±311 ppm, benefiting greatly from the significant dynamic changes in the heading.

Table 3 summarizes the minimum estimable values of the IMU systematic error states after (30). The minimum estimable values of both the accelerometer and gyroscope biases are significantly smaller than their associated observation standard errors (10 cm/s^2^ and 40 ‘/s, respectively). Of course, the impact of scale factor errors on sensor observations depends upon the range of the sensor observations. For the accelerometers, the maximum linear acceleration the test trajectory reaches is ~2.3 m/s^2^, which corresponds to an impact of 0.2–0.3 cm/s^2^ on the sensor observations for the minimum estimable accelerometer scale factor errors. For the gyroscopes, the maximum heading rate-of-change the test trajectory reaches is ~21.3°/s, which corresponds to an impact of 0.4′/s on the sensor observations for the minimum estimable z-gyro scale factor error.

#### 4.1.3. Minimum Detectable Values of Sensor Systematic Errors as Observation Outliers

**Figure 10:**
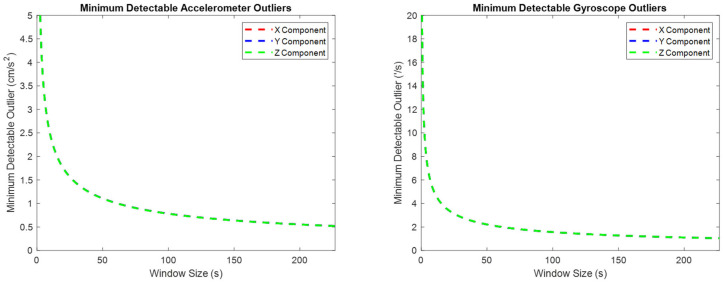
The minimum detectable observation errors for the IMU accelerometers and gyroscopes, using a variety of window sizes, ranging from 0.4 s to the entire dataset.

The minimum detectable observation errors can vary significantly depending upon the time window used to evaluate them. The minimum detectable observation errors are estimated over a variety of time windows using Equation (42) and presented in Figure 10. All three components of the accelerometer and gyroscope minimum detectable outliers have identical values for every time window used. These quantities converge well as the time window increases in size, and their final converged values are provided in Table 4.

Comparing the results from Table 3 and Table 4, the minimum detectable observation outliers in the accelerometers/gyroscopes are significantly smaller than the associated minimum estimable sensor biases. This difference draws their relative roles in the pre-analysis framework into focus:

1. The minimum estimable sensor systematic error states provide a prediction of the attainable accuracy of the system.
2. The minimum detectable observation errors provide a prediction of the system’s precision via estimating the minimum consistent observation error that would produce a detectable impact on the system’s output.

#### 4.1.4. Comparison of Pre-Analysis Results with Road Test Data

Since the pre-analysis conducted in Section 4.1.1, Section 4.1.2 and Section 4.1.3 uses a trajectory derived from a real road test dataset, the estimated minimum estimable systematic error states may be compared to the *a posteriori* minimum estimable systematic error states. The *a posteriori* values are determined via scaling the assumed sensor accuracies by their associated time-varying variances of unit weight. The process of estimating these variances of unit weight is described in detail in [24], which also shows these estimated quantities for the road test dataset used in this paper.

The road test dataset was collected from a land vehicle equipped with York University’s in-house integrated navigation system, consisting of an IMU 440 sensor operating at 100 Hz and a NovAtel OEM4 receiver operating at 1 Hz. A second NovAtel OEM4 receiver operating at 1 Hz was operated as a base station at a fixed location for the purposes of generating double-differenced GPS observations.

The plots of the minimum estimable systematic error states for the real dataset appear almost identical to the corresponding plots in Section 4.1. While this is encouraging regarding the consistency between the pre-analysis results and the road test’s results, providing these plots here would not provide meaningful insight outside of establishing this consistency. Instead, the ratio between the *a priori* and *a posteriori* minimum estimable systematic error states is provided to illustrate the actual performance of the system.

**Figure 11:**
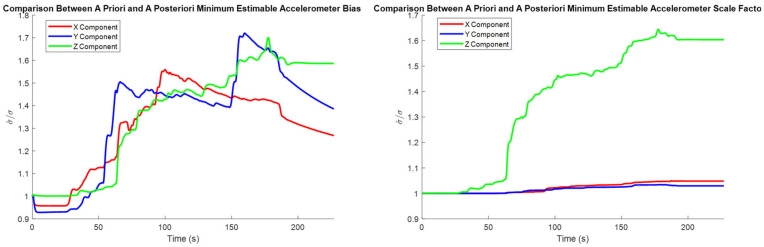
Ratio between the a priori and a posteriori standard deviations of the accelerometer bias estimates (**left**) and scale factor estimates (**right**).

**Figure 12:**
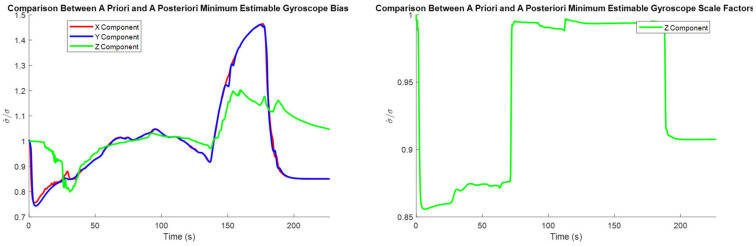
Ratio between the a priori and a posteriori standard deviations of the gyroscope bias estimates (**left**) and scale factor error estimates (**right**).

**Figure 13:**
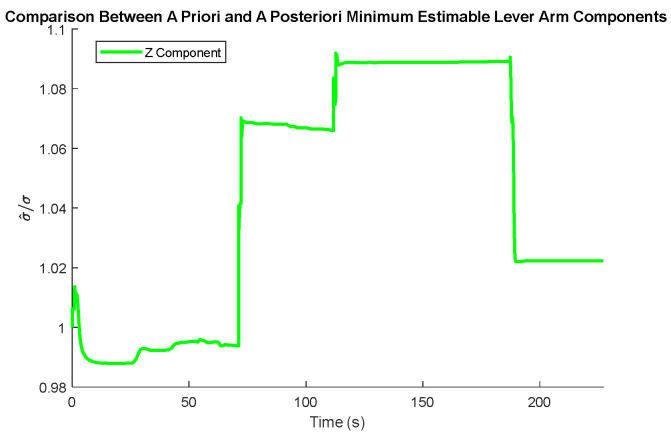
Ratio between the a priori and a posteriori standard deviations of the lever arm component estimates.

The comparison between the *a priori* and *a posteriori* minimum estimable accelerometer systematic error states is presented in Figure 11, the comparison between the *a priori* and *a posteriori* minimum estimable gyroscope systematic error states is presented in Figure 12, and the comparison between the *a priori* and *a posteriori* minimum estimable lever arm components is presented in Figure 13.

In the plots of the ratio of the *a posteriori* to *a priori* standard deviations, the calculated ratio is typically larger than one, meaning that the actual observation accuracy did not exceed the sensor specifications. Notably, the *a posteriori* minimum estimable Z-axis component of the gyroscope scale factor error outperformed the *a priori* standard deviation estimates consistently for the entire observation period.

### 4.2. Case #2: Simulated Trajectories with a Circular Motion Maneuver

Driving the vehicle carrying the positioning system in a circle is a common calibration maneuver to undertake prior to data collection procedures for an online calibrated system. With such a maneuver, there are three defining parameters that may impact the quality of the online calibration: the radius of the circular motion, the tangent velocity along the circle, and the number of times the circular motion is repeated. The number of repetitions of the maneuver is assumed to have a similar impact on all radii/linear velocity combinations, so we first identify the speed and radius that yield the lowest minimum estimable systematic errors.

#### 4.2.1. Identifying Optimum Parameters for the Circular Motion Maneuver

**Figure 14:**
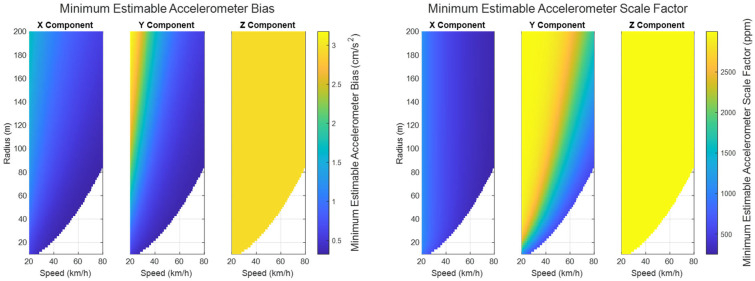
The minimum estimable values of the augmented accelerometer bias states (**left**) and scale factor error states (**right**) under circular motion (speeds ranging from 20 to 80 km/h and radii ranging from 10 to 200 m).

**Figure 15:**
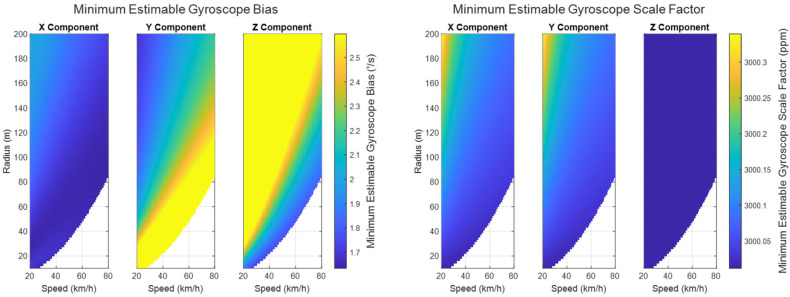
The minimum estimable values of the augmented gyroscope bias states (**left**) and scale factor error states (**right**) under circular motion (speeds ranging from 20 to 80 km/h and radii ranging from 10 to 200 m).

The minimum estimable accelerometer and gyroscope biases/scale factor errors at the end of the maneuver are shown from Figure 14 and Figure 15. The blank areas in the plots represent sets of parameters that would constitute a potentially unsafe maneuver due to the chance of skidding in a ground vehicle.

As shown in Figure 14 and Figure 15, the overall minimum estimable values for all of the augmented sensor bias states have reached a level better than 1σ of their associated sensor standard errors for both the accelerometers and gyroscopes.

The minimum estimable value of the augmented z-accelerometer’s systematic error states is not as sensitive to the speed and radius of the circular motion as the other two components are. This result is intuitive, considering that changing the parameters of the horizontal motion the system experiences does not impact the vertical acceleration of the system. Specifically, the biases and scale factor errors in the specific force measurements are better estimated when the circular motion is at a higher speed with a smaller radius.

#### 4.2.2. Directly Comparing Covariance Matrices

**Figure 16:**
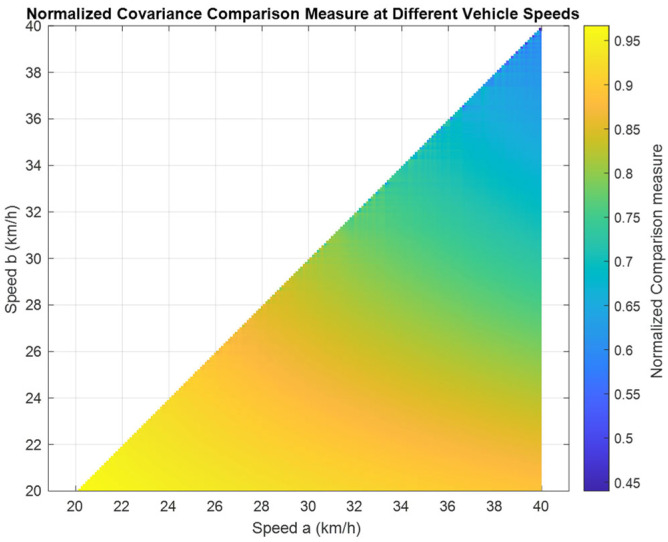
The estimated normalized covariance comparison measures for circular motion with a radius of 30 m at different vehicle speeds. The covariance comparison measure is $c_{ab}$, with $D_{a}$ determined using Speed a (X-axis) and $D_{b}$ determined using Speed b (Y-axis).

To evaluate the impact vehicle speed has on the overall quality of IMU online calibration, a pairwise comparison is conducted between different circular trajectories that have different vehicle speeds but the same circular radius of 30 m. The pairwise comparison is accomplished via the methodology provided in Section 3.3, and a plot of the normalized comparison measures is provided in Figure 16.

The normalized comparison measure indicates that higher speeds generally correspond to improvements in the overall quality of the state covariance matrix, since the normalized comparison measure is consistently positive. This improvement does yield diminishing returns as the speed increases further, and this behavior is particularly evident at speeds above 30 km/h.

## 5. Conclusions and Remarks

This manuscript proposed a novel framework of trajectory pre-analysis for the online calibration of IMU sensors using the Generic Multisensor Integration Strategy (GMIS). This allows for the performance of the following tasks in a pre-analysis environment:

1. Evaluating minimum estimable values of the augmented systematic error states for any intrinsic/extrinsic errors being modeled in the Kalman Filter.
2. Evaluating the minimum estimable length of a vector containing a group of intrinsic/extrinsic errors being modeled as part of the positioning Kalman Filter.
3. Comparing the overall accuracy of multiple potential trajectories to determine which provides an overall more reliable set of system states.
4. Evaluating the redundancy contributions of different positioning/orientation measurements for arbitrary sensor configurations over a potential trajectory.
5. Evaluating the minimum detectable values of the anticipated constant biases in the measurement and process noise vectors.

This framework for pre-analysis opens up many opportunities for rigorously evaluating different calibration maneuvers that may be integrated into the observation procedure to improve the quality of estimated IMU systematic errors. To illustrate the analysis that is possible under this framework, it was applied to the commonly-used maneuver of driving a ground vehicle-mounted positioning system in circles. In particular, this analysis was conducted using a wide variety of defining parameters for the motion, allowing for an identification of the best specific procedures to follow to maximize the information provided by this maneuver.

Additionally, this research also assessed the minimum estimable systematic error estimates for an IMU sensor for a trajectory that does not include any calibration maneuvers, to assess worst-case performance of such a system.

Evaluating the redundancy contributions of different positioning/orientation measurements has a lot of potential in evaluating non-standard positioning sensor configurations to ensure that each sensor provides the Kalman Filter with unique information.

This work represents a significant extension to the analytical capabilities of the GMIS, further establishing its robustness and flexibility and cementing its superiority in thoroughly characterizing sensor errors and conducting comprehensive error analysis.

## Figures and Tables

**Table 1:** The technical specifications of the sensor measurements used in the case studies.

Measurement	Standard Deviation	Unit
GNSS-Derived Position	±2	cm
GNSS-Derived Velocity	±3	cm/s
Accelerometer Specific Force	±10	cm/s^2^
Gyroscope Angular Rate	±40	′/s

**Table 2:** Results of evaluating the eigenvalues and eigenvectors of the normalized state covariance matrix after (23).

Dominant Eigenvector Components	Associated Eigenvalue	$\mathrm{Contribution}\;\mathrm{to}\;tr\left(D_{x}^{N}\right)$ (%)
GPS-IMU Lever Arm—X and Y	29.4769	98.256
GPS-IMU Lever Arm—Z	0.2414	0.805
GPS-IMU Lever Arm—X and Y	0.1882	0.627
Gyroscope Scale—X and Y	0.0403	0.134
Gyroscope Scale—X and Y	0.0329	0.110
Accelerometer Scale—Z	0.0022	0.007
Accelerometer Scale—X	0.0021	0.007
Accelerometer Scale—Y	0.0020	0.007
TOTAL	29.986	99.953

**Table 3:** Summary of the minimum estimable values of the IMU systematic error states (Case #1). Augmented states that were not estimated due to their lack of observability are marked with “-”.

Systematic Error State	Minimum Estimable Systematic Error		
	X Component	Y Component	Z Component
Accelerometer Bias [cm/s^2^]	1.1	1.0	0.9
Accelerometer Scale Factor Error [ppm]	1235	1455	875
Gyroscope Bias [′/s]	3.5	3.5	3.9
Gyroscope Scale Factor Error [ppm]	-	-	311
IMU-GPS Lever Arm [cm]	-	-	2.6

**Table 4:** The minimum detectable observation errors for the IMU accelerometers and gyroscopes, estimated over the entire test trajectory. Note that the detectable biases are identical across all three different sensor axes.

Observation Type	Minimum Detectable Observation Outliers		
	X Component	Y Component	Z Component
Accelerometer [cm/s^2^]	0.52	0.52	0.52
Gyroscope [′/s]	1.05	1.05	1.05

## Data Availability

The simulated trajectories used for analysis are available on request.
